# POEMS syndrome: un diagnostic à ne pas méconnaitre

**DOI:** 10.11604/pamj.2015.20.448.6353

**Published:** 2015-04-30

**Authors:** Madiha Mahfoudhi, Sami Turki, Adel Kheder

**Affiliations:** 1 Service de Médecine Interne A, Hôpital Charles Nicolle, Tunis, Tunisie

**Keywords:** Polyneuropathie, endocrinopathie, gammapathie monoclonale, polyneuropathy, endocrinopathy, monoclonal gammopathy

## Abstract

Le POEMS syndrome est une affection multi-viscérale rare qui peut s'accompagner de complications graves. Nous rapportons le cas d'un homme âgé de 48 ans qui avait une poly-neuropathie périphérique, une poly-adénopathie, des ongles blancs nacrés, une hypothyroïdie et une gammapathie monoclonale. L’évolution était marquée par l'aggravation des signes neuroloqiques. Des cures de thalidomide-dexaméthasone ont été administrées sans aucune amélioration clinique. Le patient était rapidement décédé. Le but de ce travail est d'insister sur le polymorphisme clinique et le pronostic variable de cette entité.

## Introduction

Le POEMS syndrome est une affection systémique multi-viscérale rare associant une polyneuropathie (P), une organomégalie (O), une endocrinopathie (E), une gammapathie monoconale (M) et des lésions cutanées (S). Des tableaux atypiques peuvent s'accompagner parfois d'atteintes viscérales graves notamment neurologiques et rénales. Le polymorphisme des manifestations cliniques au cours de ce syndrome rend le diagnostic difficile retardant ainsi la prise en charge.

## Patient et observation

C’était un homme âgé de 48 ans qui a présenté en 2005 un accident vasculaire cérébral avec découverte d'un diabète de type 2. L’évolution était marquée par l'apparition deux ans après d'une poly- neuropathie hyperalgique des membres inférieurs et une aréflexie diffuse avec à l’électromyogramme une neuropathie démyélinésante sensitivo-motrice des 4 membres prédominant aux membres inférieurs. Six mois après, le malade a développé une poly-adénopathie cervicale, axillaire, médiastinale et abdominale sans hépato-splénomégalie. Le patient avait des ongles blancs nacrés finement striés. La biopsie d'un volumineux ganglion axillaire gauche a trouvé un ganglion réactionnel en évolution adipeuse. Le bilan biologique a révélé une hypertriglicéridémie, une hypothyroïdie, une hyperoestrogènémie responsable d'une gynécomastie bilatérale et d'un dysfonctionnement érectile avec une gammapathie monoclonale de type IgA lambda. Le bilan radiologique était normal. Le bilan biologique a objectivé une thrombocytose. Le myélogramme a montré des plasmocytes de morphologie normale à 4%. Le diagnostic du POEMS syndrome a été retenu. Des cures de thalidomide-dexaméthasone ont été administrées sans amélioration clinique. L’évolution était marquée par l'aggravation rapide de l’état neurologique avec apparition d'une diplopie transitoire, de phosphènes, d'une baisse de l'acuité visuelle, d'un tremblement d'attitude avec un syndrome vestibulaire, de trouble de la marche, d'une aggravation de la neuropathie sensitivo-motrice des quatre membres et d'un accident vasculaire cérébral compliqué de crises convulsives généralisées. L’évolution était rapidement fatale.

## Discussion

Le syndrome POEMS est plus fréquent au Japon selon Dispenzieri et al [1] et Nakanishi et al [2]. La prédominance masculine est rapportée. L’âge moyen de survenue se situe entre 40 et 50 ans. Typiquement le syndrome POEMS associe les cinq manifestations principales annoncées dans le sigle POEMS, comme c'est le cas des deux présentations. Certains auteurs exigent la présence de deux critères majeurs (neuropathie, gammapathie monoclonale) avec un critère mineur parmi les critères suivants: lésion ostéocondensante, maladie de Castleman, organomégalie, ‘dème (périphérique, pleurésie, ascite), endocrinopathie (surrénale, hypophyse, parathyroïde, thyroïde, diabète), manifestations cutanées (hyperpigmentation, hypertrichose, angiomes, ongles blancs, oedème papillaire). Ces critères ont été proposés par l’équipe de Dispenzierri et al [1]. La polyneuropathie est l’élément constant, retrouvé dans 100% des cas dans la série de Nakanishi et al [2]. C'est une polyradiculonévrite chronique se manifestant par des paresthésies des pieds (71%), des douleurs (12%), des troubles de la marche (17%), une aréflexie diffuse (68%) et des troubles de la sensibilité profonde (78%). Dans la mise au point de Soubrier (2007), la polyneuropathie est le plus souvent révélatrice. Dans notre observation, l'atteinte neurologique était inaugurale et d'aggravation rapide, caractérisée par des accidents vasculaires cérébraux récurrents qui seraient liés à l'hyperviscosité par thrombocytose. Une thrombocytose est présente chez 54 à 88% des patients, une polyglobulie chez 12 à 20% comme il a été démontré dans les études de Dispenzieri et al [1] et Nakanishi et al [2]. Il a été rapporté que l’œdème papillaire bilatéral est la manifestation ophtalmologique la plus fréquente, généralement asymptomatique [3]. L'atteinte rénale est observée dans 10% des POEMS syndromes comme ont illustré les travaux de Dispenzieri et al [1] et Soubrier et al [3]. Gherardi et al [4], Soubrier et al [5] et D′Souza et al [6] ont évoqué le rôle de l'interleukine 6 et des facteurs de croissance et notamment le vasculor endothelial growth factor (VEGF) dans la genèse des lésions endothéliales et de l’œdème mésangial. La glomérulonéphrite membrano-proliférative et la prolifération cellulaire mésangiale ont étés rapportées plusieurs travaux [1, 2]. En dehors des cinq manifestations principales du syndrome POEMS, la présentation clinique est très polymorphe, comme il a été démontré dans la plupart des études [1–5] pouvant comporter: un syndrome fébrile avec altération de l’état général, une insuffisance rénale, des AVC, une diarrhée, des épanchements pleuraux, une HTAP primitive et une insuffisance cardiaque. Soubrier. M a insisté sur le fait que les patients ayant une neuropathie périphérique inexpliquée doivent bénéficier d'une électrophorèse et d'une immunoélectrophorèse à la recherche d'un composant monoclonal orientant vers ce diagnostic pour réduire le retard diagnostique et améliorer la réponse thérapeutique de ces lésions neurologiques [7]. Le traitement par la thalidomide ou le lénalidomide, prescrits par Tomás et al [8], ont donné de bons résultats avec diminution du taux de VEGF et de la neuropathie. L'atteinte neurologique chez notre patient était sévère, la thalidomide n’était pas efficace. L'association melphalan-dexaméthasone a donné de bons résultats dans certains cas comme il a été présenté dans la revue de Méndez-Herrera et al [9]. La greffe de moelle serait indiquée dans les formes réfractaires à la corticothérapie, la chimiothérapie et la radiothérapie. Le bévacizumab, anticorps monoclonal anti-VEGF, a amélioré de façon spectaculaire 2 patients mais en a aggravé un autre [7]. L’évolution fatale chez notre patient serait en rapport avec le retard diagnostique et thérapeutique.

## Conclusion

Le syndrome POEMS est une maladie grave qui peut engager le pronostic fonctionnel et vital par le retentissement sur l’état général et les atteintes viscérales surtout rénale, neurologique et cardiaque. La précocité diagnostique et la réponse au traitement sont les facteurs pronostiques essentiels.
